# Recovery of cranial nerve neuropathies after LINAC-based stereotactic radiosurgery for benign cavernous sinus meningioma

**DOI:** 10.1007/s11060-024-04783-3

**Published:** 2024-08-01

**Authors:** Tehila Kaisman-Elbaz, Philip Blumenfeld, Marc Wygoda, John Feldman, Yigal Shoshan

**Affiliations:** 1 Department of Neurosurgery, Hadassah Hebrew University Medical Center, Jerusalem, Israel; 2 Radiotherapy Institute, Hadassah Hebrew University Medical Center, Jerusalem, Israel

**Keywords:** Cavernous sinus meningioma, Stereotactic radiosurgery, Cranial nerve neuropathy, Recovery, Improvement, Volume

## Abstract

**Purpose:**

Cranial Nerve Neuropathies (CNNs) often accompany Cavernous Sinus Meningioma (CSM), for which Stereotactic Radiosurgery (SRS) or fractionated stereotactic radiotherapy (FSR) are established treatments. This study assesses CNNs recovery in CSM patients treated with LINAC, offering insight into treatment effectiveness.

**Methods:**

This study was conducted on 128 patients with CSM treated with LINAC-based SRS/FSR between 2005 and 2020 at a single institution. 46 patients presented with CNNs. The study analyzed patients’ demographics, clinical parameters, SRS/FSR treatment characteristics, post-treatment CNNs recovery duration, status, and radiological control on their last follow-up.

**Results:**

The median follow-up duration was 53.4 months. Patients were treated with SRS (n = 25) or FSR (n = 21). The mean pretreatment tumor volume was 9.5 cc decreasing to a mean end-of-follow-up tumor volume was 5.1 cc. Radiological tumor control was achieved in all cases. CNN recovery was observed in 80.4% of patients, with specific nerve recoveries documented as follows: extra-ocular nerves (43.2%), trigeminal nerve (32.4%), and optic nerve (10.8%). A higher CNNs recovery rate was associated with a smaller pre-treatment tumor volume (p < 0.001), and the median time-to-improvement was 3.7 months. Patients with tumor volumes exceeding 6.8 cc and those treated with FSR exhibited prolonged time-to-improvement (P < 0.03 and P < 0.04 respectively).

**Conclusions:**

This study suggests that SRS/FSR for CSM provides good and sustainable CNNs recovery outcomes with excellent long-term radiological control. A higher CNNs recovery rate was associated with a smaller pre-treatment tumor volume. while shorter time-to-improvement was identified in patients treated with SRS compared to FSR, particularly in those with small pre-treatment tumor volume.

## Introduction

Stereotactic radiosurgery (SRS) and fractionated stereotactic radiotherapy (FSR) are well-established treatment modalities for symptomatic cavernous sinus meningioma (CSM) [1–4] with numerous studies demonstrating their ability to achieve excellent tumor control and low complication rates when compared to surgical resection [5–9]. Many patients diagnosed with CSM are symptomatic, presenting with progressive cranial nerve neuropathies (CNNs) [1, 8]. The recovery patterns of CNNs following SRS/FSR are not well-established and further insight into predictors of CNNs recovery rates, the extent of recovery, and time-to-improvement is needed to better inform treatment decisions and patient expectations.

The recovery rate of CNNs following treatment for CSM varies widely among studies, with rates ranging from 20% to 69.3% [7, 8, 10] and requiring long-term follow-up to confirm permanence [8, 10]. Recovery rates for specific cranial nerves also differ between studies and are occasionally conflicting. For example, recovery rates for facial pain and numbness have been reported as high as 40–76% [11] in some studies, while others demonstrate a 30% recovery rate for extra-ocular nerves (EONs) and trigeminal deficits [12]. Additionally, studies have demonstrated that diplopia improved in 45.5% of cases [13], while recovery rates for trigeminal and EON deficits ranged from 25 to 76% and 24–57%, respectively. The use of mixed patient populations in studies, including both symptomatic and asymptomatic individuals, contributes to the uncertainty of recovery patterns for strictly symptomatic CSM patients [8, 12, 14].

The choice between SRS and FSR is typically based on factors such as tumor volume and/or its proximity to critical structures. A 2018 meta-analysis showed that the major outcomes of CNNs following SRS/FSR were independent of the fractionation schema [6]. However, the data on LINAC-based SRS/FSR is lacking. Similarly, in an additional meta-analysis [8], only two LINAC-based studies were qualified to be included [12, 15]. Despite the well-established efficacy of both SRS and FSR, the infrequency of reporting on LINAC-based SRS/FSR in the literature compared to the Gamma Knife radiosurgery series [11, 14, 16], may impact the strength of evidence [6].

This study focused specifically on symptomatic CSM patients treated with LINAC-based SRS/FSR and aims to define their course of illness and provide further insight into CNNs’ time-to-improvement and recovery patterns.

## Patients and methods

### Patients’ selection process

This retrospective study was approved by the institutional review board and included 128 consecutive patients diagnosed with CSM between the years 2005–2020 and subsequently treated with SRS/FSR at the Hadassah Hebrew University Medical Center, Jerusalem, Israel.

To identify eligible patients, a thorough search was conducted, and inclusion criteria were established as follows: 1) brain MRI scans demonstrating CSM; 2) patients with CNNs; 3) patients subsequently treated with either SRS/FSR. For patients without a histological diagnosis, a diagnosis of meningioma was suggested by two independent neuro-radiologists and following a neurosurgery/neuro-oncology tumor board discussion. Exclusion criteria were: 1) pathological diagnosis of other cavernous sinus lesion; 2) patients with histologically proven WHO grade II/III meningiomas; 3) asymptomatic patients; 4) prior microsurgery-induced permanent CNNs; 5) previous brain irradiation; 6) insufficient follow-up or limited documentation. Out of the entire cohort of CSM patients treated with SRS/FSR, 46 patients met the inclusion/exclusion criteria, and their data were collected and analyzed.

### Cohort characterization

The selected patients’ cohort was identified by a comprehensive data search and retrieval of their neuro-ophthalmologists, neurosurgery, and neuro-oncology clinic follow-up medical records. Demographics, disease course, and clinical features were documented, including presenting symptoms, onset and duration, recovery status, and duration following SRS/FSR treatment. Patients’ files were also searched for post-treatment new CNNs. High-resolution MRIs during treatment and at the last follow-up were reviewed for all patients. Pre- and post-treatment tumor volume were measured at the pre-and post-SRS/FSR treatment MRI scan by Brainlab Elements Cranial SmartBrush software.

### LINAC-based SRS and FSR treatment

Patients were treated with either SRS/FSR based on a multidisciplinary discussion as recommended by consensus guidelines [1]. Until 2016, a rigid stereotactic frame head fixation for single fraction SRS treatment, and a rigid thermoplastic face-specific mask were used for fractionated treatments. As of 2016, a rigid thermoplastic face-specific mask was used for both SRS and FSR patients. High-definition CT scans were obtained followed by Axial 3D T1-gadolinium MRI, 0.5 mm slice thickness sequences. Image data sets were fused using treatment planning and tumor volume and organs at risk were defined. Target volumes were defined as Gross Tumor Volume (GTV) encompassing the entire tumor volume without additional margin for Planning Target Volume (PTV). Treatment planning was performed using dynamic conformal arc therapy and/or Intensity Modulated Radiotherapy techniques and approved by the treating physician. The decision to treat with SRS/FSR was made based on the distance of the superior border of the tumor to the optic pathway (minimal distance of 2 mm) and/or the tumor volume (exceeding 9 cc).

Twenty-five (54.3%) of the patients were treated with single fraction SRS [(n = 21, median marginal radiation dose of 12.8 Gy (12–13 Gy), or 2–5 fractions, cumulative total dose of 18–25 Gy, n = 4)], to the 80% isodose line (IDL). 21 (45.7%) of the patients were treated with full fractionation FSR protocol of 27 daily fractions (five days a week) of 1.8 Gy (marginal dose) to the 90% IDL (cumulative total dose of 54 Gy).

Importantly, the maximum point dose was calculated for the optic apparatus and brainstem which were visualized on the image dataset, and it was possible to define them volumetrically. The dose constraints utilized were the accepted TG-101 constraints. Identifying and delineating CN III, IV, V, and VI within the CSMs was not possible, and therefore, their maximum point dose could not be assessed.

Treatment delivery was performed by our LINAC-based platform available at the time (2005–2015—Varian DBX with Brainlab M3; 2016–2020—Truebeam Novalis STx with ExacTrac X-Ray). Upon completion of treatment, patients were given dexamethasone and scheduled for 3-month follow-up brain MRI scans and neurosurgical clinic visits. Further follow-up care was based on patients’ symptomatology and imaging results at a follow-up visit. Radiosurgery treatment parameters were collected and documented.

### SRS/FSR clinical and radiological outcomes evaluation

Upon data retrieval completion, patients’ information was summarized and analyzed. Treatment-related parameters were calculated, including time-to-treatment defined as the time elapsed between the diagnosis to the SRS/FSR treatment date, follow-up duration, radiological tumor volumes, and tumor control rates based on pre-and post-treatment tumor volumes measurements. CNNs recovery-related parameters were assessed during routine neurosurgical outpatient follow-up visits and determined both by patient report and neurological examination. Cranial nerve II function evaluation was based on records from neuro-ophthalmology clinic including visual acuity, fundoscopy, visual fields, and optical coherence tomography. Criteria for CN II deficit status were based upon visual field and acuity test results. Improvement status and recovery duration (time-to-improvement) defined as the time gap between the treatment date to the first documented improvement of the pre-treatment CNN and post-treatment new CNs deficits onset rates were determined.

Tumor volumes, tumor response, and control were assessed for individual patients, measuring the difference between pre- and post-treatment tumor volumes in brain MRI scans. Tumor control was defined as radiological tumor volume stabilization (up to 10% of post-treatment volume reduction) or tumor volume regression measurement (10% or higher of post-treatment volume reduction), consistent with previous assessments in literature [13].

### Statistical analysis

The study population was divided into subgroups based on various characteristics such as SRS/FSR treatment, recovery status, duration, and pace. The Mann–Whitney U test was used to compare the subgroups’ characteristics and statistical significance was determined when the p-value was less than 0.05. Kaplan Meier curves were used to assess time-to-event and were compared using the log-rank test. Pearson correlation coefficient R was also calculated to assess the interdependence of different parameters.

## Results

### Patients’ demographics and clinical characteristics

The demographic and clinical characteristics of the study population are summarized in Table 1. Notably, 18 of the 46 patients (38.3%) underwent prior CSM resection, and 2 patients underwent resection of another intra-cranial meningioma. These patients had a confirmed histopathology diagnosis of WHO grade I meningioma. The remaining patients with CSM who did not undergo tumor resection were considered to be WHO grade I, as their clinical behavior and radiological appearance were consistent with this diagnosis.

**Table 1:** Patients’ Main Demographics and Clinical Characteristics. As demonstrated in the table, most of the patients’ cohort were females. 60% of the patients were treated within 1 year of symptoms onset and were longitudinally followed up for a median duration of 53.4 months. Most of the patients demonstrated deficits of extraocular nerves and cranial nerve V

	Parameter	Value (Range)
Demography	Patients’ cohort (n)	46
	Gender (female)	78.7
	Mean age (years)	51.8 (19.1–75.7)
Clinical characteristics	Median follow-up duration (months)	53.4 (3.9–190.4)
	Median symptoms onset-to-treatment time (months)	2.9 (1–69.4)
	Treated within 1 year (%)	60
	Treatment within more than 1 year (%)	23
	N/A	17
Cranial nerves type deficits (%)	Cranial nerve II	17
	Extraocular nerves (EONs) deficits	36.1
	Cranial nerve V	25.5
	Combined cranial nerves deficits	19.1

### Radiological tumor response

**Fig. 1:**
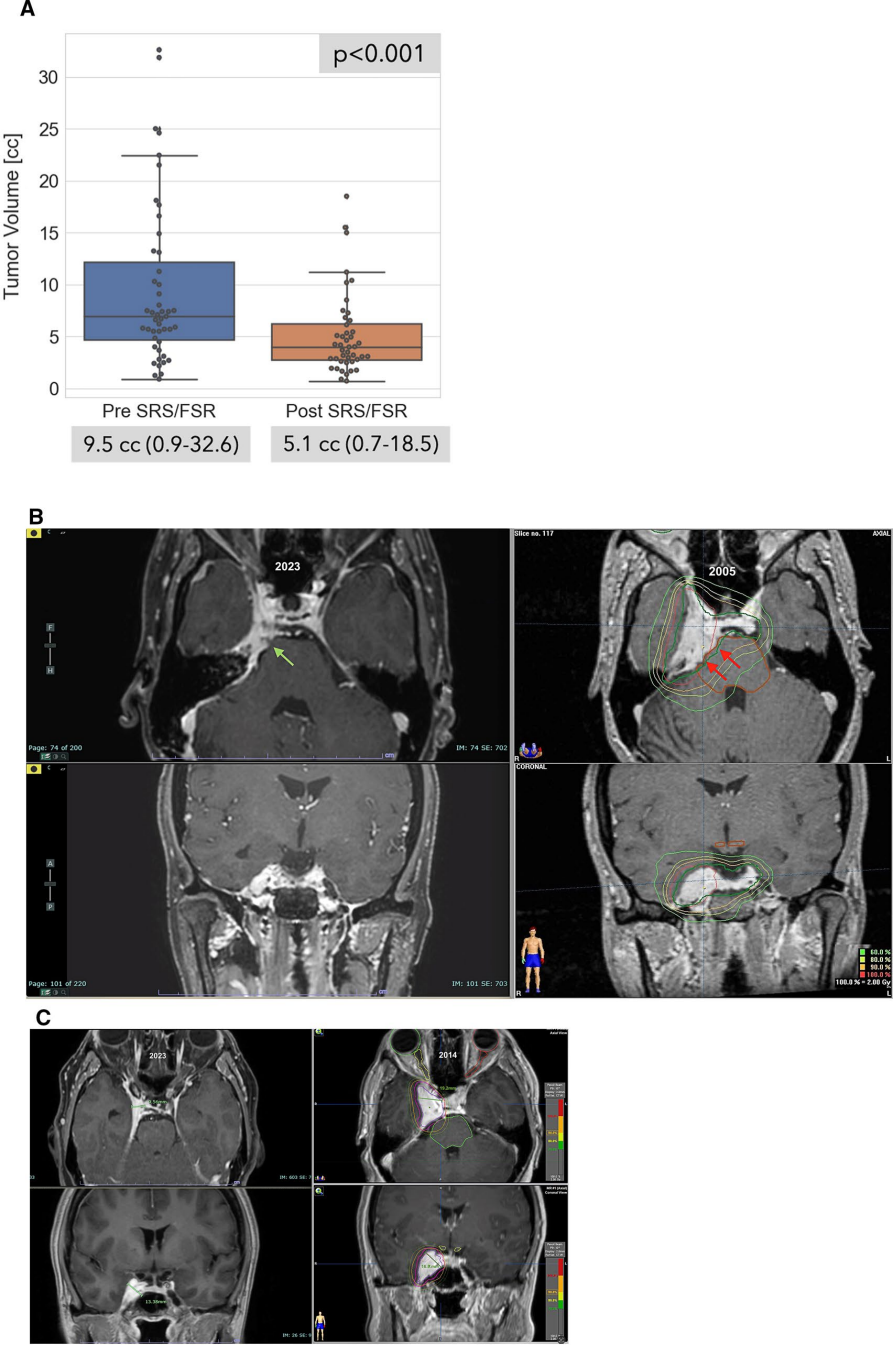
Radiological Tumor Response. Graphic depiction of mean pre- and post-SRS/FSR tumor volumes (**A**). As demonstrated in the graph, the mean pre-SRS/FSR treatment tumor volume was 9.5 cc compared to the mean post-treatment tumor volume which was 5.1 cc. A 100% tumor control rate was therefore identified. A representative case of a patient with CSM who was treated in the year 2005. A significant tumor reduction was detected at the last follow-up MRI in 2023. The red and green arrows represent the tumor interface with the brainstem, which is markedly reduced (**B**). Another representative case of a CSM patient who was treated in the year 2014 and a follow-up scan that was conducted in 2023. The bar within the CSM mass boundaries represents the calculated tumor diameter and the difference detected between the two time points. Tumor volume reduction was 57%. The figures also contain the radiation dose distribution and treatment isodose lines as well as the optic apparatus and brainstem markings (**C**)

Figure 1A presents the main treatment-related radiological assessment. The mean post-treatment tumor volume was 5.12 cc (0.7–18.5) which significantly decreased compared to the mean pre-treatment tumor volume (9.5 cc, p < 0.001). Tumor volume reduction was detected in 80.1% of the patients, with a mean volume reduction of 50%. Tumor volume stabilization was detected in 20% of the patients. No patient in this study cohort exhibited radiological post-treatment tumor volume progression. Therefore, the 5-year tumor control rate of this cohort was defined as 100%. Two representative cases of post-SRS/FSR tumor volume reduction throughout patients’ follow-up are presented in Fig. 1B, C.

### CNNs recovery characterization

**Fig. 2:**
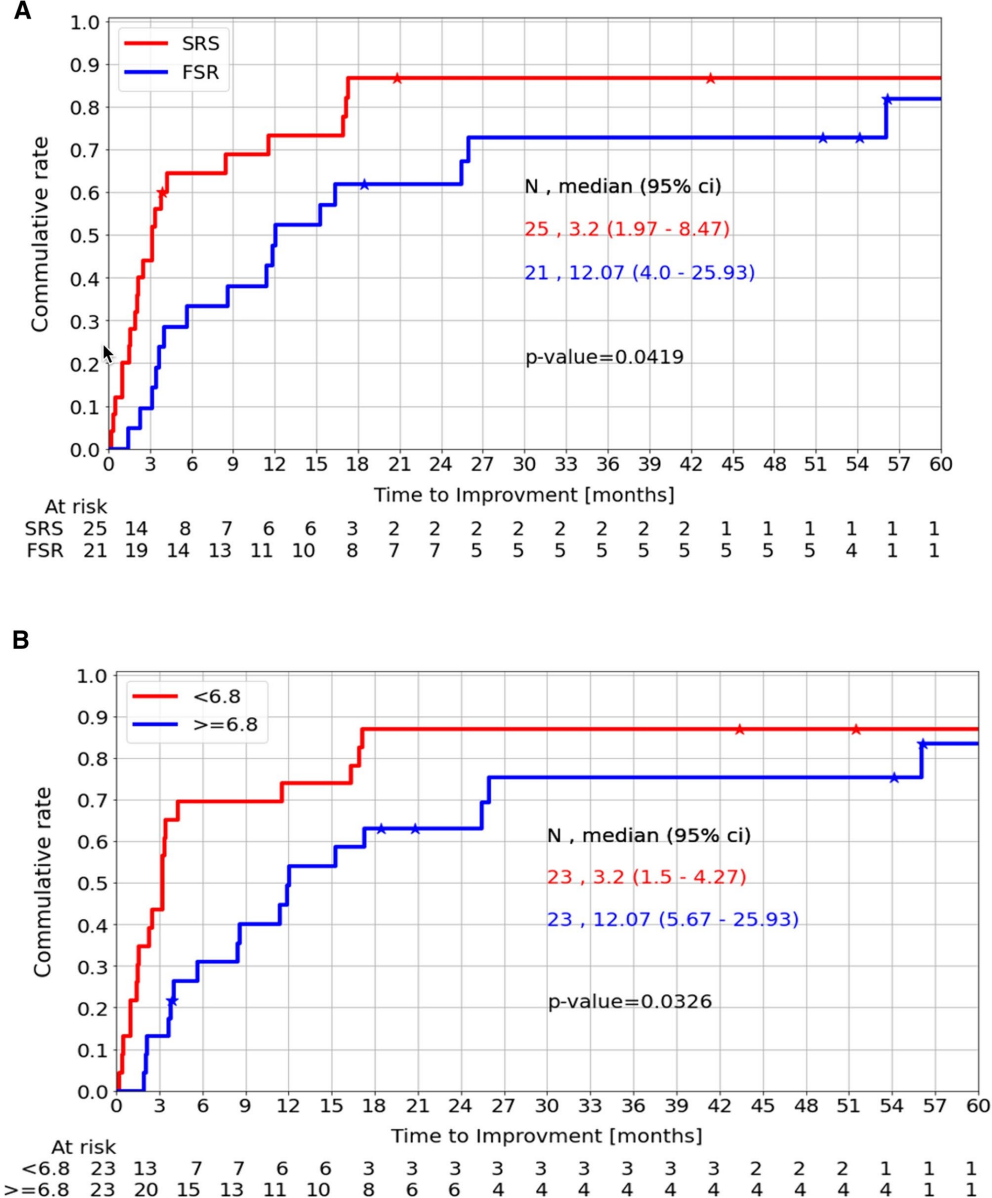
Time-to-improvement dependency on SRS/FSR treatment (**A**) and pre-treatment tumor volume (**B**). SRS-treated patients demonstrated significantly enhanced time-to-improvement compared to FSR-treated patients (3.2 vs. 12.07 months). A similar result was demonstrated regarding median pre-treatment tumor volumes below 6.8 cc

The overall recovery rate of CNNs was not significantly different between the FSR and SRS-treated patients (Table 2A). However, the median time-to-improvement of both groups differed significantly between the two groups (3.2 vs. 12.07 months, respectively, p = 0.04), as shown in Fig. 2A. Pearson correlation analysis revealed a strong positive association between the follow-up period and longer time-to-improvement (r = 0.83) in the FSR-treated patients. Furthermore, pre-treatment tumor volume was found to similarly influence time-to-improvement, with patients with pre-treatment tumor volume < 6.8 cc experiencing shorter time-to-improvement periods compared to those with larger pre-treatment tumor volumes (3.2 vs. 12.07 months, respectively, p = 0.03), as shown in Fig. 2B. CNNs, including EONs, showed a similar distribution in both treatment groups and there were no significant differences in full recovery rates between these subgroups.

**Table 2:** A Comparison of SRS/FSR Treatment Effect (A). Notably, patients undergoing SRS treatment had shorter follow-up and median time-to-treatment compared to patients treated with FSR. Mean pre-treatment tumor volumes and post-treatment tumor volume reduction also differed significantly, as did the median time-to-improvement. Post-treatment Cranial Nerves Neuropathies Recovery Pattern Outcomes (B). A significant number of patients demonstrated improvement following SRS/FSR treatment (80.4%), within a median time-to-improvement of 3.7 months. 56.7% of the patients demonstrated full recovery and the extraocular cranial nerves, as well as cranial nerve V, showed an enhanced recovery rate. Small numbers of patients demonstrated new post-treatment cranial nerve deficits

A			
Parameter	SRS (n = 25)	FSR (n = 21)	Statistical significancy
Mean range	52.1	51.4	p = 0.48
Gender (female, %)	72	86.4	p = 0.73
Median follow-up duration, months	28.6	64.1	**p < 0.001**
Median time-to-treatment, months	2.1	5.3	**p < 0.037**
Mean pre-treatment tumor volume (cc)	6.3	13.1	**p < 0.001**
Mean post-treatment tumor volume reduction (%)	30.7	52.4	**p < 0.001**
CNN improvement rate (%)	84	77.3	p < 0.71
Median time-to-improvement (n = 37), months	n = 21, 2.5 months	n = 16, 10 months	**p < 0.004**

B		
	Parameter	Value (Range)
CNN overall recovery rate	Improvement rate	80.4%
	Median time-to-improvement (months)	3.7 (0.2–56.1)
CNN recovery status (%)	Full	56.7
	Partial	43.2
	None	19.5
CNN recovery rate, by CNs (%)	Cranial nerve II	10.8
	Extraocular nerves (EONs)	43.2
	Cranial nerve V	32.4
	Combined cranial nerves deficits	13.5
Post-treatment new deficit (%)	Trigeminal neuropathy	7
	Lingual neuropathy	2

Table 2B presents the main findings on CNNs recovery patterns. Among the 46 patients who underwent SRS/FSR treatment, 37 (80.4%) demonstrated any CNNs recovery at a median time-to-improvement of 3.7 months (range, 0.2–56.1). Of these patients, 56.7% showed full recovery of their CN function while 43.2% defined as partial CNNs recovery. Specifically, 8 (17%) patients had isolated cranial nerve II deficits, and only one patient experienced improvement. Of the 12 patients (25.5%) with trigeminal neuropathy presenting with facial paresthesia (tingling) and sensory loss (none with trigeminal neuralgia), four (32.4%) experienced an improvement in the intensity of their paresthesia. Of the 36.1% of patients with extraocular muscles deficit, 43.2% experienced CN improvement (Tables 1 and 2B).

Further analysis was conducted to explore recovery rates in the patient cohort, by subdividing them into those who recovered (n = 37) and those who did not (n = 9), as shown in Table 3A. Demographic characteristics and follow-up duration were similar between the two groups, as were the time-to-treatment parameters. While tumor volume reduction did not differ significantly between the groups, pre-treatment tumor volume was significantly higher in the non-recovered group (14.4 cc vs. 8.1 cc, p = 0.03). Although most patients in the non-recovered group were treated with FSR, this parameter by itself did not have statistical significance, nor was the involvement of EONs deficit related to the rate of recovery.

**Table 3:** Characterization of Cranial Nerves Neuropathies’ Recovery Rate and Duration. A comparison of recovered versus not recovered patients’ characteristics (A) as well as patients’ duration of recovery (B). As demonstrated in these two tables, the main parameters shown to impact patients’ recovery patterns were the mean pre-treatment tumor volume, the median time-to-treatment, and FSR treatment

	Recovered (n = 37)	Not recovered (n = 9)	Statistical significance
*A*			
Mean age (years)	50.5	50.2	p = 0.36
Median time-to-treatment, months, (mean)	2.9 (10.4)	2.8 (12.9)	p = 0.5
Mean pre-treatment tumor volume (cc)	8.1	14.4	**p = 0.03**
Mean tumor volume reduction (%)	38.9	49.4	p = 0.15
FSR (yes, %)	43.2	55.5	p = 0.71
Median follow-up duration, months (mean)	53.4 (61.2)	51.5 (55.8)	p = 0.38
*B*			
Duration recovery (months), median = 3.67	≤ 3.67 (0.4–3.7) (n = 19)	> 3.67 (3.8–56.1) (n = 18)	
Mean age (years)	53.8	48.5	p = 0.26
Median time-to-treatment, months (mean)	2.2 (8.6)	6.1 (12.1)	**p = 0.05**
Mean pre-treatment tumor volume (cc)	5.3	10.7	**p = 0.002**
Mean tumor volume reduction (%)	31	46.4	p = 0.054
FSR (yes, %)	21.1	66.7	**p = 0.02**
Median follow-up duration, months (mean)	36 (71.3)	56.9 (50.6)	p = 0.083

Pearson correlation analysis of the parameters characterizing the recovered patients’ group indicated that time-to-improvement is linked to a longer follow-up period (r = 0.6). Specifically, in the FSR-treated patient’s analysis, the time-to-improvement and follow-up period was even stronger (r = 0.71). However, this correlation was not found in the SRS-treated group (r = 0.052). Furthermore, an analysis of the parameters of the non-recovered patients’ group showed that time-to-treatment was negatively correlated with age at diagnosis (r = −0.65) while other parameters were not found to be significantly correlated.

Table 3B presents the time-to-improvement data about selected clinical and radiological parameters. The median time-to-improvement for the entire cohort of recovered patients was 3.67 months which was used as the cutoff to subdivide the cohort into fast- and slow-recovering groups. Large pre-treatment tumor volumes were found to significantly prolong the time-to-improvement recovery (5.3 vs. 10.7 cc, p = 0.002) as well as FSR treatment compared to SRS (p = 0.02). Pearson correlation analysis demonstrated a strong correlation between patients’ time-to-improvement was follow-up duration in the slow-recovery group of patients (r = 0.74). No significant correlations were found among the parameters examined in the fast-recovering group.

## Discussion

This study provides evidence supporting the efficacy of both SRS and FSR as treatment options for CSM [3, 17]. Various studies indicated high long-term tumor control rates (tumor volume stability or tumor regression [13]) of over 90% and low complication rates of approximately 10% [17, 18]. The results of this study support these findings with 100% tumor control rate, 8.2% new CNNs onset rate, and 80.4% of CNNs recovery rate measured in symptomatic CSM patients treated with either delivery method.

However, the literature on the recovery of CNNs following SRS for CSMs is somewhat limited, and despite recent consensus statements by EANS [1] and ISRS [5], there is still a lack of guidance and recommendations regarding this topic. Available data on recovery rates is inconsistent with wide-ranging rates of 20–69% [7, 8, 10], possibly due to the lack of criteria for assessing CNNs recovery [10] or the inclusion of mixed symptomatic and asymptomatic patient populations in the studies or due to relatively small sample sizes. This lack of clear evidence can pose difficulties in accurately informing patients of the potential treatment benefits of SRS/FSR before the procedure.

Several factors have been identified as potentially contributing to the recovery of CNN, including upfront radiosurgery [19], treatment within the first year since CNN presentation [12, 13], sporadic disease, WHO grade I histology, and the number, type, and severity of the CNNs [8, 12]. On the other hand, age, gender, pre-treatment tumor volume, and radiological tumor regression, were not found to affect the recovery rate [6, 10]. The favorable high recovery rates following SRS/FSR treatment in this study may have been influenced by the fact that most patients were treated within 12 months of symptoms onset and were classified as WHO grade I. The study findings support previous reports that demographics and tumor volume reduction rate are not associated with patient recovery.

This study found that pre-treatment tumor volume was smaller in the group of patients who recovered, measuring 8.1 cc compared to 14.4 cc, and was associated with a faster time-to-improvement. This finding is consistent with a recent study by Amelot et al. [20], which demonstrated that smaller CSMs induce more severe symptoms such as cranial nerve palsy or persistent trigeminal neuralgia, resulting in earlier diagnosis and treatment. In contrast, larger CSMs may cause a more insidious clinical course and induce minor and indefinite symptoms such as headaches, intermittent diplopia, or paresthesia, leading to delayed treatment. Indeed, in our study, patients who recovered quickly were characterized by reduced-volume tumors and had a time-to-treatment of 2.2 months, whereas the slow-recovering patients had significantly larger tumors and a time-to-treatment of 6.1 months. The study also demonstrated that the speed of recovery was faster in the SRS compared to the FSR group. We may suspect that this difference is attributed to the slightly higher BED (assuming alpha/beta ratio 3.5) in the SRS group (91.86 Gy BED) compared to FSR (84.86 Gy BED).

Previous studies have investigated the post-radiosurgery radiological tumor response of CSMs, but the findings have been inconsistent. Some studies, such as Leroy et al. [6], and Chung et al. [17], have demonstrated that FSR stabilizes the tumor, while SRS induces regression, which is attributed to different radiobiologic effects on the irradiated tumor tissue and varying follow-up durations. However, other studies, such as Correa et al. [10], have reported no significant difference in tumor regression between SRS and FSR-treated patients, despite a long-term follow-up of 73 months. It should be noted that the study by Correa et al. had a larger mean treatment tumor volume for FSR-treated patients (25.39 cc) compared to SRS-treated patients (8.25 cc), which could impact the assessment of tumor regression since changes in larger tumors may be more challenging to detect [23].

Our study found that both SRS and FSR were effective in inducing significant radiological tumor regression, but the regression magnitude differed between the two treatment methods. FSR-treated CSMs resulted in a greater reduction in tumor volume (52.4%) than SRS-treated CSMs (30.7%). However, it should be noted that the follow-up duration in the SRS-treated patients was shorter (28.6 months) than that in the FSR-treated patient group (64.1 months), which may be due to the relatively rapid recovery observed in the SRS-treated patients (time-to-improvement demonstrated of 2.5 vs. 10 months in FSR-treated patients).

It is important to acknowledge that the reason for the difference in follow-up duration between the two groups was not fully investigated in this study. However, it may arise in part since in the first years of the radiosurgery service operation in our institution, radiosurgery treatments were mostly given as FSR rather than SRS. This tendency was attributed to cautiousness and probably led to the longer follow-up demonstrated in the FSR group. As the radiosurgery service was further established and experience accumulated, more patients were treated with SRS.

Our study’s findings align with previous research that has suggested a link between follow-up duration and time-to-improvement [8, 12, 24] in treating meningiomas with radiosurgery. Additionally, it is generally observed that most meningiomas tend to show signs of regression within the first few months following radiosurgery treatment [21]. However, the significant difference in radiological tumor volume reduction observed between SRS and FSR treatment, coupled with the finding that larger tumors have a slower recovery rate, may suggest that the radiation response delivered by FSR played a role in this difference. Alternatively, the initial large size of the tumor could have impacted its behavior and response rate to treatment. However, due to the small sample size in our study, it is challenging to differentiate between these factors with certainty.

It is important to note that the clinical behavior of CSMs can be unpredictable, and recovery rates can vary among different benign pathologies involving the cavernous sinus [20, 21]. For example, Umekawa et al. [24], reported that CSM-induced CNNs may recover to a lesser extent than those resulting from other cavernous sinus benign pathologies, possibly due to the infiltrative nature of CSM cells and their aggressive, unpredictable behavior [20, 25], which can lead to high recurrence rates [26]. In our study, 19.1% of patients with CNNs did not recover, and no statistically significant parameter was associated with this group of patients. Amelot et al. [20], conducted a recent study on the natural history of CSMs and observed a consistent non-improvement CNNs rate of approximately 21% in medically treated patients, who did not undergo upfront SRS/FSR treatment. These findings may suggest that roughly 20% of symptomatic CSM patients with CNNs may not experience recovery, irrespective of the treatment approach or other factors.

To summarize, this study suggests that both SRS and FSR can effectively control CSMs, but they differ in terms of tumor regression and recovery rates. FSR may result in greater tumor volume reduction but may require a longer time for improvement than SRS. The recovery rate of CNNs is not significantly different between the two treatments, and approximately 20% of patients may not recover regardless of the treatment modality or other parameters. Large CSMs treated with FSR and patients with combined CNs deficits and isolated optic neuropathies may be associated with lower CNNs recovery rates. In contrast, patients with trigeminal or EONs deficits may exhibit higher recovery rates. Rapid symptom alleviation may have a positive impact on the final recovery status of CNNs, and the use of steroids before treatment initiation is suggested [20] but requires further investigation.

Indeed, this study has limitations that should be taken into consideration. As a retrospective study conducted in a single institution, the results may not be generalizable to other patient populations or treatment centers. In addition, the relatively small sample size may limit the statistical power and generalizability of the findings. The study design also did not allow for a direct comparison between SRS and FSR, as the treatment modality was chosen based on various factors such as tumor volume and location. Lastly, as with all retrospective studies, the possibility of selection bias cannot be excluded. Therefore, larger prospective studies that specifically evaluate symptomatic CSMs are necessary to confirm the results of this study and establish uniform evaluation criteria. Multicenter studies would also provide more robust data and allow for a direct comparison between SRS and FSR. Additionally, studies that evaluate the long-term effects of SRS and FSR on CSMs, including tumor control and symptom alleviation, would provide valuable information for clinical decision-making.

## Data Availability

No datasets were generated or analysed during the current study.
